# Glutathione S-Transferase Omega-2 and Transforming Growth Factor-*β*1 Polymorphisms in Iranian Glaucoma Patients

**DOI:** 10.1155/2021/1061650

**Published:** 2021-11-23

**Authors:** Shahram Bamdad, Fatemeh Sanie-Jahromi, Marzieh Alamolhoda, Nasrin Masihpour, Mohammad-Hossein Karimi

**Affiliations:** ^1^Poostchi Ophthalmology Research Center, Department of Ophthalmology, School of Medicine, Shiraz University of Medical Sciences, Shiraz, Iran; ^2^Transplant Research Center, School of Medicine, Shiraz University of Medical Sciences, Shiraz, Iran

## Abstract

**Background:**

To investigate the association of glutathione s-transferase omega 2 (GSTO2) (142N > D) and transforming growth factor-*β*1 (TGF-*β*1) (869T > C) gene polymorphisms on the pathogenesis of two common types of glaucoma (including primary open-angle glaucoma (POAG) and chronic angle-closure glaucoma (CACG)) in the Iranian population.

**Methods:**

A total of 100 glaucoma patients (60% males and 40% females with an age mean ± SD of 34.66 ± 14.25 years; 56 cases of POAG and 44 cases of CACG) were enrolled in this study. GSTO2 (142N > D) and TGF-*β*1 (869T > C) polymorphisms were evaluated by PCR-based methods in patients and controls.

**Results:**

At locus GSTO2 (142N > D), the odds of ND genotype with respect to DD and NN genotypes were 1.55 and 2.08 times higher in POAG and CACG patients compared to those of patients in the control group (95% CI_1_: 0.80–2.98; 95% CI_2_: 1.00–4.33) which was statistically significant in CACG patients. However, the odds of DD and NN genotypes against the reference genotype in two patients group were not statistically significant as compared to those of patients in the control group. There was a significant association between the ND genotype and male patients (OR = 2.28, 95% CI: 1.06–4.92). The analysis of TGF-*β*1 (869T > C) polymorphisms showed no significant difference between the genotypes of TGF-*β*1 (869T > C) polymorphisms in patients and control groups; however, the CT genotype of TGF-*β*1 significantly differed between female controls and patients (OR = 0.42, 95% CI: 0.18–0.96).

**Conclusion:**

The presented results revealed that there was a significant association between the ND genotype of GSTO2 and the pathogenesis of glaucoma. Furthermore, this genotype can be considered as a sex-dependent genetic risk factor for the development of glaucoma. In contrast, the CT genotype of TGF-*β*1 is suggested to be a protective genetic factor against the pathogenesis of glaucoma.

## 1. Background

Glaucoma is a continuous developing disease determined by the apoptosis of the retinal ganglion cells. This feature leads to the excavation of the optic nerve head, visual field loss, and blindness probably [1]. It is believed that glaucoma is an ocular neuropathy with a specific pattern of destroying the optic nerve disk and visual field, which may be due to multiple socioenvironmental conditions [2]. Pathophysiologically speaking, glaucoma is mainly caused by increased intraocular pressure (IOP) which ultimately causes compression and damage to the optic nerve, loss of neural tissue, and vision loss [3]. Recent studies have shown that glaucoma is associated with disruption of the optic nerve head extracellular matrix [4] and oxidative stress [5].

There is persuasive evidence indicating that genetics has a significant role in the pathogenesis of glaucoma. Glutathione S-transferase (GSTs) and transforming growth factor-*β* (TGF-*β*) are the important genes under study in this case [6].

GSTs (EC: 2.5.1.18) are present in various ocular structures, such as the aqueous humor, ciliary body, and lens [7]. Glaucoma seems to be associated with GSTs in that oxidative damage by reactive oxygen species is remarkably increased in glaucoma [8]. GSTs are a part of the drug-metabolizing phase II enzyme family that detoxify endogenous electrophilic xenobiotics and inactivate the end-products during oxidative stress. They are a family of genes with different classes scattered in the cytosol, mitochondria, and microsomes. GST omega (GSTO) is a class of cytosolic GSTs and possesses a novel role in thiol transferase and reduction reactions which are not performed by other GST classes [9, 10]. In population studies, two GSTO gene polymorphisms, GSTO1 (140A > D) and GSTO2 (142N > D), have been recognized [11]. Although the association of polymorphism of GST family genes has been investigated with specific eye diseases including cataracts [12, 13] and senile macular degeneration, no report is available on the association of GSTO2 (McKusick no. 612314) and glaucoma.

Another important pathogenic factor for glaucoma is TGF-*β*. Overexpression of TGF-*β* in the trabecular meshwork and increased deposition of ECM have been reported in glaucoma patients [4]. TGF-*β* is an important cytokine involved in different cellular processes such as the production of the extracellular matrix. There are three isoforms of TGF-*β* (TGF-*β*1 through TGF-*β*3) in mammals [14]. TGF-*β*1 (McKusick no. 190180) is believed to have a crucial role in the induction of ECM production. Several polymorphism loci and single nucleotide polymorphisms (SNPs) have been investigated in the TGF-*β*1 gene [15]. The 869T > C polymorphism is one of the best-known polymorphisms in the TGF-*β*1 gene and is known to be associated with several diseases [16–18] and eye disorders [19]. It has been shown that 869T > C polymorphism is located in the coding sequence of TGF-*β*1 and leads to altered expression and/or function of TGF-*β* [18]. The association of other TGF-*β*1 polymorphisms such as 509C > T with glaucoma has been surveyed in some nations, yielding different results [20, 21]. Yet, there is no report on the effect of TGF-*β*1 (869T > C) polymorphism on glaucoma.

This study aimed to identify the association of TGF-*β*1 (869T > C) and GSTO2 (142N > D) polymorphisms with two common types of glaucoma (including primary open-angle glaucoma (POAG) and chronic angle-closure glaucoma (CACG)) in the Iranian population.

## 2. Methods

Study group: in this project, we randomly selected 100 glaucoma patients (60% males and 40% females with a mean ± SD age of 34.66 ± 14.25 years), including 56 cases of POAG and 44 cases of CACG. No other type of glaucoma was included in this study, and it is a coincidence that the mean age of patients in this study is lower compared to that in other studies. The control group consisted of 106 individuals (47.16% males and 52.83% females) who were matched in terms of age and sex with the patient group. The male to female ratio (M/F) was 60/40 (1.5) in the patients' group and 50/56 (0.89) in the control group. All patients were Iranian and were selected from the Motahhari glaucoma clinic after an examination by a glaucoma specialist. Written informed consent was obtained and approved by the ethics committee of Shiraz University of Medical Sciences. The inclusion and exclusion criteria for each type of glaucoma were detailed elsewhere [22].

Records were reviewed, a general medical history was taken, and complete slit-lamp and fundus examinations were conducted. Patients had Humphrey automated white on white stimulus static perimetry. Optical coherence tomography (OCT) was carried out. The control group consisted of 106 Iranian individuals. They were matched for sex and age with the patient group, and no symptomatic, metabolic, genetic, or ocular disorders on an extensive questionnaire about family history, past medical problems, and current health status were reported.

### 2.1. Sample Collection and DNA Extraction

Peripheral blood (5 ml) was collected in EDTA tubes from all participating individuals after obtaining their written consent. DNA extraction was performed using the DMP DNA isolation kit from Gentra Systems (Minneapolis, MN) and stored in aliquots at −20°C.

### 2.2. Genotyping

Gene polymorphisms were evaluated by polymerase chain reaction using a thermal cycler (Techne, Genius, UK). Primers, product size, restriction enzyme, and PCR methods are summarized in Table 1. The genotype of GSTO2 (142N > D) was determined by the PCR-restriction fragment length polymorphism (RFLP) method, as described previously [23]. The PCR-RFLP products corresponding to NN, ND, and DD genotypes were separated by electrophoresis on 2% agarose gel. The amplification refractory mutation system- (ARMS-) PCR method was carried out for the identification of TGF-*β*1 (869T > C) polymorphism, as described previously [24]. A *β*-globin gene primer was applied as an internal control. After PCR, the products were digested by a restriction enzyme, and the amplified products were monitored by agarose gel electrophoresis and ethidium bromide staining.

### 2.3. Statistical Analysis

Genotype frequencies were calculated in patient and control groups by direct gene counting. The chi-square test (*χ*^2^) was conducted to examine the Hardy–Weinberg equilibrium (HWE) of data, and the associations between genotype distribution in patients and controls were determined. Statistical analyses were performed using SPSS 16.0 (SPSS Inc., released in 2007 (SPSS for Windows, Version 16.0. Chicago, IL, USA)). Odds ratios (ORs) and 95% confidence intervals (CIs) were calculated to assess the association between genotypes in patients and control groups, using MedCalc software version 8.0. All *P* values of less than 0.05 were considered as statistically significant.

## 3. Results

The demographics of the study population, including gender, age, and glaucoma staging (based on AAO classification) is summarized in Table 2. The present study evaluated the association of GSTO2 (142N > D) and TGF-*β*1 (869T > C) polymorphisms with glaucoma of two common types (POAG and CACG). GSTO2 (142N > D) and TGF-*β*1 (869T > C) polymorphisms of patients were identified by gel electrophoresis of PCR products following PCR-RFLP and ARMS-PCR.  Table 3 shows the genotypes distribution of GSTO2 (142N > D) and TGF-*β*1 (869T > C) polymorphisms and OR for each genotype in the patient vs. control groups. Of note, the frequency of the TGF-*β*1 (869T > C) polymorphism and GSTO2 (142N > D) polymorphisms reported in our study is in line with the other literature, at least in the normal Iranian population [18, 25, 26]. The percentage of ND and CT genotypes polymorphism at GSTO2 (142N > D) and TGF-*β*1 (869T > C) locus was higher in the patient and control groups compared to the other genotypes; however, there was no statistically significant association between the genotypes and patient and control groups (*χ*^2^_(2)_ = 4.23, *P*=0.12 at GSTO2 (142N > D) and *χ*^2^_(2)_ = 1.31, *P*=0.52 at TGF-*β*1 (869T > C)). The distribution of genotype frequencies was consistent with HWE in both groups. The OR of each genotype was measured considering the corresponding genotype compared to the sum of other genotypes (reference genotype) in each locus. According to Table 2, at locus GSTO2 (142N > D), the odds of the ND genotype with respect to DD and NN genotypes were 1.55 and 2.08 times higher in POAG and CACG patients compared to those in the control group (95% CI_1_: 0.80–2.98; 95% CI_2_: 1.00–4.33) which was statistically significant in CACG patients. However, the odds of DD and NN genotypes against the reference genotype in two patients group were not statistically significant as compared to those in the control group (DD genotype: OR_1_ = 0.71, OR_2_ = 0.54; 95% CI_1_: 0.35–1.44, 95% CI_2_: 0.25–1.14; NN genotype: OR_1_ = = 0.75, OR_2_ = 0.38; 95% CI_1_: 0.29–1.93, 95% CI_2_: 0.11–1.38 in POAG and CACG patients compared to the control group). Moreover, the ratio of odds of TT, CT, and CC to the reference genotype was not statistically significant in POAG and CACG patients group vs. control group at locus TGF-*β*1 (869T > C).

The profile of the GSTO2 genotype in glaucoma patients and controls concerning their gender is summarized in Table 4. The distribution of the genotypes in each gender was not significantly different between the patient and control groups (all *P* values >0.05). As represented by the data, the odds of the ND genotype of GSTO2 were significantly higher in male patients compared to those in their corresponding controls (OR_1_ = 2.28, 95% CI: 1.06–4.92). Furthermore, the odds of the CT genotype against the CC and TT genotypes were lower in female patients compared to those in the female control group at TGF-*β*1 locus (OR_2_ = 0.42, 95% CI: 0.18–0.96).

## 4. Discussion

To the best of our knowledge, this is the first report on the association of GSTO2 (142N > D) polymorphism with glaucoma patients in the Iranian population considering different genders. Previous studies have emphasized the association between GST polymorphisms in other classes of GSTs (such as mu and theta) and the risk of eye diseases including cataracts [12, 13] and senile macular degeneration [27]. In a study conducted by Juronen et al., GSTM1 polymorphism seemed to be associated with more likely progression of PAOG [28]. In 2007, it was reported that the GSTM1-positive genotype and GSTT1 null genotype or the combination of both may be correlated with the higher risk of POAG in the Turkish population [29]. Izzotti et al. reported that POAG was associated with the GSTM1 null genotype in the Italian population [30]. Also, the GSTM1 null genotype has been found recently to be related to a higher risk of POAG [31]. Juronen et al. were the first who examined the possible association between the polymorphic GST genotypes and adult-onset POAG [28]. They suggested a similar relationship between the GSTM1 genotype and POAG incidence [28]. In contrast, in 2003, it was reported that there was no evidence to confirm the association between GSTM1 polymorphism and glaucoma in the Swedish population [32]. Our results demonstrated that the ND genotype of GSTO2 could be considered as a genetic risk factor for the development of glaucoma, especially in males. Our results showed that the frequency of the heterozygous genotype is significantly associated with this disease. Such an association between the heterozygous genotype and a specific phenotype has been reported in other studies [33]. However, in order to confirm this observation, we performed random sample sequencing of PCR products which proved the data. We also used control genotypes in the genotyping run.

TGF-*β* is one of the cytokines with significantly elevated levels in the anterior chamber of glaucomatous eyes. It has been shown that TGF-*β* has a direct effect on intraocular pressure. It is suggested that enhanced intraocular pressure is due to a complex interaction with the trabecular meshwork, resulting in decreased aqueous humor outflow [34]. Recent research indicates that TGF-*β* causes extracellular matrix protein (ECM) to increase in the trabecular meshwork, besides its elevated level in the aqueous humor of glaucomatous patients [4]. TGF-*β*2 polymorphism has been under the focus of previous studies regarding its effect on glaucoma pathology. Recently, TGF-*β*1 has attracted the attention of population genetics researchers, and the association of TGF-*β*1 different polymorphisms and glaucoma has been surveyed. Sripriya et al. have demonstrated that the TGF-*β*1-509C > T polymorphism might not be correlated to POAG [20]. In this study, we surveyed the association of TGF-*β*1 (869T > C) polymorphism and glaucoma from both POAG and CACG patients in Iran. In the same line with previous results, our results showed that TGF-*β*1 (869T > C) polymorphisms did not differ significantly in glaucoma patients and healthy controls. What distinguishes this study from the previous ones was that the relationship between TGF-*β*1 polymorphism was investigated in different genders. According to our results, the CT genotype of TGF-*β*1 showed a remarkable difference between the patient and control groups in females. These findings suggest that females with the CT genotype at locus TGF-*β*1 are significantly at lower risk for glaucoma disease.

## 5. Conclusions

In conclusion, the represented results showed that the ND genotype of GSTO2 could be considered as a genetic risk factor for the development of glaucoma. The ND genotype of GSTO2 can be a candidate for sex-dependent genetic risk factors. Moreover, the CT genotype of TGF-*β*1 was shown to be a protective genetic factor against glaucoma in females.

### 5.1. Strength and Limitations

Since previous studies have highlighted the importance of the association of GSTO2 and TGF-*β*1 genes with the pathogenesis of glaucoma, this study aimed to survey the association of two important previously overlooked polymorphisms of these genes with two common types of glaucoma. Evidence from this study showed that genotype GSTO2 (142N > D) has a significant association with the pathogenesis of glaucoma in southern Iran, which can be an important finding in predicting the pattern of the disease and preventive care in people with this genotype. The present study is valuable in that there are no reports of an association between TGF-*β* 869T > C and GSTO2 (142N > D) with glaucoma. The small number of samples studied, the limited sampling time, and younger age of the glaucoma patients are of the limitations of this study. It is suggested that a similar study be conducted with a larger number of samples over a longer period of time in the future.

## Figures and Tables

**Table 1:** The primer, PCR methods, restriction enzymes, and product size for TGF-*β*1 and GST-O2.

Locus	Primers	Product size	Method (restriction enzyme)
GSTO2 (142N > D)	(Sense primer) 5′-AACCCTCCTAAAGCACCC-3′ (Antisense primer) 5′-GCCTGTGAAAGCTGGTGTTAG-3′	NN: 185bp ND: 185bp, 122bp, 63bp DD: 122bp, 63bp	RFLP-PCR (MboI)

TGF-*β*1 (869T > C)	(Sense primer) common:5-TCCGTGGGATACTGAGACACC-3 (Antisense primer) C allele: 5-GCAGCGGTAGCAGCAGCG-3 (Antisense primer) T allele: 5-AGCAGCGGTAGCAGCAGCA-3	CC: 110bp CT: 241bp, 110bp TT: 241bp	ARMS-PCR

**Table 2:** Demographics of the study population.

Glaucoma patients	*n*	Male	Female	Mean age (±SD)	Glaucoma staging (AAO classification)			Clinical demographics^*∗*^	
	100	60	40	34.66 ± 14.25	Mild	Moderate	Severe	Mean IOP (±SD) (mmHg)	Mean CCT (±SD) (*μ*m)
POAG	56	34	22	34.13 ± 12.9	20	21	15	19.22 ± 3.45	527 ± 23.81
CACG	44	26	18	35.33 ± 15.81	19	16	9	17.17 ± 2.02	510 ± 15.93
Control group	106	50	56	38.26 ± 12.71	N/A			16.31 ± 2.51	530 ± 18.22

CCT: central corneal thickness, IOP: intraocular pressure. ^*∗*^The clinical demographics reported in patients are during the period they have been treated with timolol, latanoprost, and Brimogan.

**Table 3:** The frequencies of GSTO2 (142N > D) and TGF-*β*1 (869T > C) genotypes in POAG, CACG patients, and control groups.

Locus	Genotype	POAG, N (%)	CACG, N (%)	Control, N (%)	OR_1_	OR_2_	95% CI_1_	95% CI_2_
GSTo2 (N142D)	DD	16 (28.6)	12 (27.3)	38 (35.8)	0.71	0.54	0.35–1.44	0.25–1.14
	ND	33 (58.9)	29 (65.9)	51 (48.1)	1.55	**2.08** ^*∗*^	0.80–2.98	1.00–4.33
	NN	7 (12.5)	3 (6.8)	17 (16.0)	0.75	0.38	0.29–1.93	0.11–1.38

TGF-*β* (T869C)	TT	12 (21.4)	13 (29.5)	29 (27.4)	0.72	1.11	0.34–1.56	0.51–2.42
	CT	25 (44.6)	18 (40.9)	54 (50.9)	0.78	0.67	0.40–1.42	0.33–1.36
	CC	19 (33.9)	13 (29.5)	23 (21.7)	1.85	1.51	0.90–3.81	0.68–3.35

**N:** number, **OR:** odds ratio, **CI:** confidence interval. **OR**_**1**_**and OR**_**2**_: odds of each genotype to reference in POAG and CACG patient groups/the odds of each genotype to reference for the control group. ^*∗*^*P* value< 0.05. **CI**_**1**_**and CI**_**2**_: confidence interval of OR_1_ and OR_2_ in POAG and CACG patients groups.

**Table 4:** The frequencies of GSTO2 (142N > D) and TGF-*β*1 (869T > C) genotypes in patients with glaucoma and controls after grouping according to gender.

Locus	Genotype	Patients, male, *N* (%)	Control, male, *N* (%)	Patients, female, *N* (%)	Control, female, *N* (%)	*χ*_1_^2^ (*P* value)	OR_1_	95% CI _1_	*χ*_2_^2^ (*P* value)	OR_2_	95% CI _2_
GSTO2 (142N > D)	DD	20 (33.33)	23 (46)	8 (20)	15 (26.78)	4.77 (0.09)	0.59	0.27–1.27	1.06 (0.59)	0.68	0.26–1.81
	ND	35 (58.33)	19 (38)	27 (67.5)	32 (57.14)		**2.28** ^*∗*^	1.06–4.92		1.56	0.67–3.63
	NN	5 (8.33)	8 (16)	5 (12.5)	9 (16.07)		0.48	0.15–1.56		0.75	0.23–2.42

TGF-*β*1 (869T > C)	TT	14 (23.33)	10 (20)	11 (27.5)	13 (23.21)	0.79 (0.67)	1.22	0.49–3.04	5.06 (0.08)	1.25	0.49–3.18
	CT	28 (46.66)	21 (42)	15 (37.5)	33 (58.92)		1.21	0.57–2.58		**0.42** ^*∗*^	0.18–0.96
	CC	18 (30)	19 (38)	14 (35)	10 (17.85)		0.70	0.32–1.55		2.48	0.96–6.36

*χ*_1_^2^ and *χ*_2_^2^ are the associations between genotype distribution and patients/control groups in male and female. **OR**_**1**_ and **OR**_**2**_ are the odds of each genotype to reference in the patients group/the odds of each genotype to reference for the control group in male and female. **CI**_**1**_ and **CI**_**2**_ are the confidence interval of OR_1_ and OR_2_ in each gender group. ^*∗*^*P* value < 0.05.

## Data Availability

The data that support the findings of this study are available from the corresponding author, M-H K, upon reasonable request.

## Abbreviations

AAO: American Academy of Ophthalmology
ARMS: Amplification refractory mutation system
CACG: Chronic angle-closure glaucoma
CIs: Confidence intervals
ECM: Extracellular matrix protein
GSTO2: Glutathione s-transferase omega 2
HWE: Hardy–Weinberg equilibrium
IOP: Intraocular pressure
OCT: Optical coherence tomography
OR: Odds ratio
POAG: Primary open-angle glaucoma
RFLP: Restriction fragment length polymorphism
SNPs: Single-nucleotide polymorphisms
TGF-*β*1: Transforming growth factor-*β*1.
